# Administration of hydrogen-rich water prevents vascular aging of the aorta in LDL receptor-deficient mice

**DOI:** 10.1038/s41598-018-35239-0

**Published:** 2018-11-14

**Authors:** Masumi Iketani, Kanako Sekimoto, Tsutomu Igarashi, Mayumi Takahashi, Masaki Komatsu, Iwao Sakane, Hiroshi Takahashi, Hideo Kawaguchi, Ritsuko Ohtani-Kaneko, Ikuroh Ohsawa

**Affiliations:** 1 Biological Process of Aging, Tokyo Metropolitan Institute of Gerontology, Tokyo, Japan; 2 Department of Life Sciences, Toyo University, Gunma, Japan; 3 Department of Ophthalmology, Nippon Medical School, Tokyo, Japan; 4 Central Research Institute, ITO EN Ltd., Makinohara, Japan

## Abstract

The main cause of arteriosclerosis is atherosclerosis in the aorta. Atherosclerosis is recognized as a chronic inflammatory condition that begins with the dysfunction or activation of arterial endothelium. Low-density lipoprotein (LDL) and especially its oxidized form play a key role in endothelial dysfunction and atherogenesis. Recent studies showed that senescent cells are involved in the development and progression of atherosclerosis, and eliminating senescent cells suppresses the senescence-associated secretory phenotype. We previously reported that molecular hydrogen-rich water (HW) has antioxidant and anti-inflammatory effects in numerous diseases. Here, we used LDL receptor-deficient mice fed a high-fat diet (HFD) for 13 weeks as a model for atherosclerosis and evaluated the effects of continuous administration of HW. The numbers of endothelial cells in the atheroma expressing the senescence factors p16^INK4a^ and p21 decreased in HFD-fed mice given HW compared with HFD-fed mice given control water. Furthermore, macrophage infiltration and *Tnfα* expression in the atheroma were also suppressed. These results suggest that vascular aging can be suppressed by HW.

## Introduction

Atherosclerosis is the most frequently occurring form of arteriosclerosis, and it can lead to severe diseases such as myocardial infarction and cerebral infarction. During the development of atherosclerosis, low-density lipoprotein (LDL) first enters into the intima of blood vessels that have been damaged by high blood pressure and/or hyperglycemia and becomes oxidized. Monocytes then infiltrate the endothelium and differentiate into macrophages. When macrophages mainly absorb large quantities of oxidized LDL, they become foam cells, forming atheromas and inducing local inflammation^1,2^. Atheromas contain senescent cells (SCs) and are thought to be related to atherogenesis, which is associated with the progression of atherosclerosis^3^. SCs cannot divide, due to arrest of the G1/S phase checkpoint. The cyclin-dependent kinase (CDK) inhibitors p16^INK4a^ and p21 promote cellular senescence and play a role specifically in vascular EC senescence^4,5^. Senescence-associated secretion phenotype (SASP) factors such as inflammatory cytokines are secreted by SCs and act on healthy peripheral tissues^6,7^. These findings indicate that SCs are involved in the biological alterations that accompany aging and many age-related diseases, including arteriosclerosis^8–10^. Recent studies using LDL receptor-deficient mice (*Ldlr*^−/−^ mice) as a model of atherosclerosis showed that the selective removal of active foam cells expressing senescence-associated β-galactosidase (SA-β-Gal) using the Bcl-2 family protein inhibitor ABT-263 perturbs the proatherogenic environament^11^.

Molecular hydrogen (H_2_) reduces highly reactive oxygen species (ROS) including the hydroxyl radical and peroxynitrite, and functions as an antioxidant and anti-inflammatory agent^12^. The routes of H_2_ administration in animal models and human clinical studies are roughly classified into three types: inhalation of H_2_ gas, drinking H_2_-rich water (HW), and injection of H_2_-dissolved saline^13^. Inhalation of H_2_ gas ameliorates acute diseases such as ischemia-reperfusion injury and graft injury in several organs. Injecting H_2_-dissolved saline is as effective against ischemia-reperfusion injury as the inhalation of H_2_ gas^13,14^. Although drinking HW, the most convenient method of H_2_ administration, results in lower H_2_ concentrations in the blood than inhalation of H_2_ gas, it can be more effective at reducing oxidative stress and inflammation^13^. For example, drinking HW ameliorated symptoms of Parkinson’s disease in a rat model, whereas both H_2_ gas inhalation and lactulose-induced production of intestinal H_2_ did not^15^. We recently reported that pre-administration of HW was sufficient to attenuate septic inflammation in mice, strongly indicating that the therapeutic effects of HW derive from a mechanism distinct from the direct reduction of ROS by H_2_^16^.

In our previous study, H_2_ administration prevented arteriosclerosis in apolipoprotein E-deficient (*Apoe*^−/−^) mice, a model of spontaneous atherosclerosis development^17^, suggesting that H_2_ administration suppresses vascular aging. Here, we examined whether drinking HW affects cellular senescence in atherosclerosis using *Ldlr*^−/−^ mice.

## Results

### Drinking HW decreased senescence markers in *Ldlr*^−/−^ mice fed a high-fat diet

**Figure 1:**
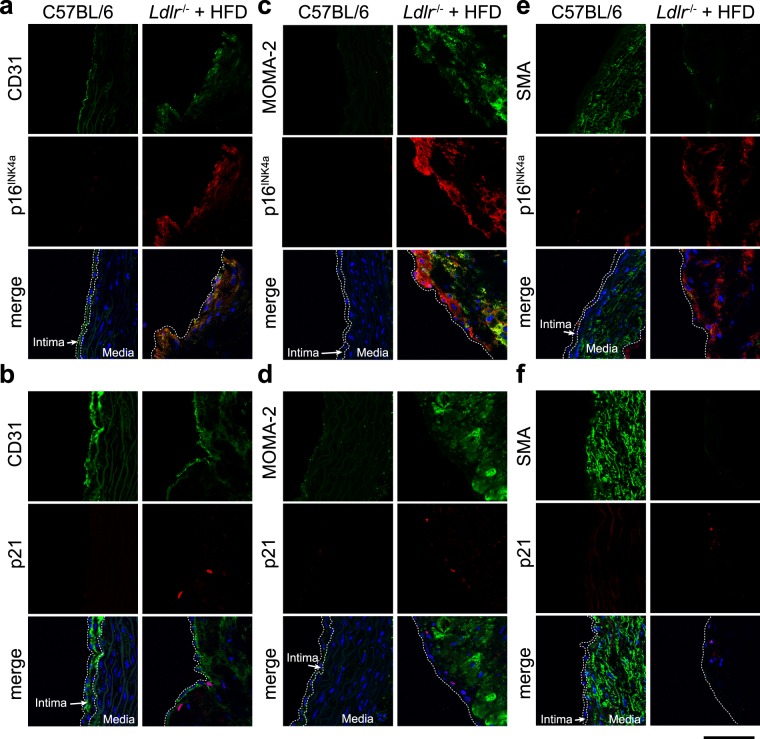
Expression of p16^INK4a^/p21 was detected in CD31-positive ECs in the aortas of *Ldlr*^−/−^ mice. Immunohistochemical staining of the ascending aorta in control-fed C57BL/6 and HFD-fed *Ldlr*^−/−^ mice with anti-p16^INK4a^ (**a**,**c**,**e**) and anti-p21 (**b**,**d**,**f**) antibodies. The aorta was coimmunostained with anti-CD31 (an ECs marker), MOMA-2 (a monocyte/macrophage marker), or anti-SMA (a marker for smooth muscle cells), and counterstained with Hoechst 33342 (blue). Dotted lines indicate the borders between blood vessel layers (intima and media). Scale bar: 50 μm.

To induce SCs in atheromas, *Ldlr*^−/−^ mice on a B6.129S7-*Ldlr*^*tm1Her*^*/*J background were fed a high-fat diet (HFD) consisting of 36% calories from fat. To determine whether drinking HW eliminates SCs, age-matched, 10-week-old *Ldlr*^−/−^ mice were randomly divided into five groups: 1) regular chow diet (control diet) with control water; 2) control diet with HW; 3) HFD with control water; 4) HFD with HW; and 5) HFD with ABT-263, which was used as a positive control. Age-matched C57BL/6 J mice were fed a control diet and provided control water in the same facility over the same time period, as an additional control. All mice were sacrificed at 13 weeks from the start of the experiment. Immunohistochemical staining of the ascending aorta with antibodies against the senescence markers p16^INK4a^ and p21 was performed to determine whether HW ameliorates HFD-induced cellular senescence in *Ldlr*^−/−^ mice. As previously reported, p16^INK4a^ and p21 were expressed in CD31-positive endothelial cells (ECs) in atheromas in HFD-fed *Ldlr*^−/−^ mice (Fig. 1a,b). Monoclonal rat anti-macrophages/monocytes antibody (MOMA-2)-positive macrophages and α-smooth muscle actin (SMA)-positive smooth muscle cells mostly did not stain with the p16^INK4a^ and p21 antibodies (Fig. 1c–f).

**Figure 2:**
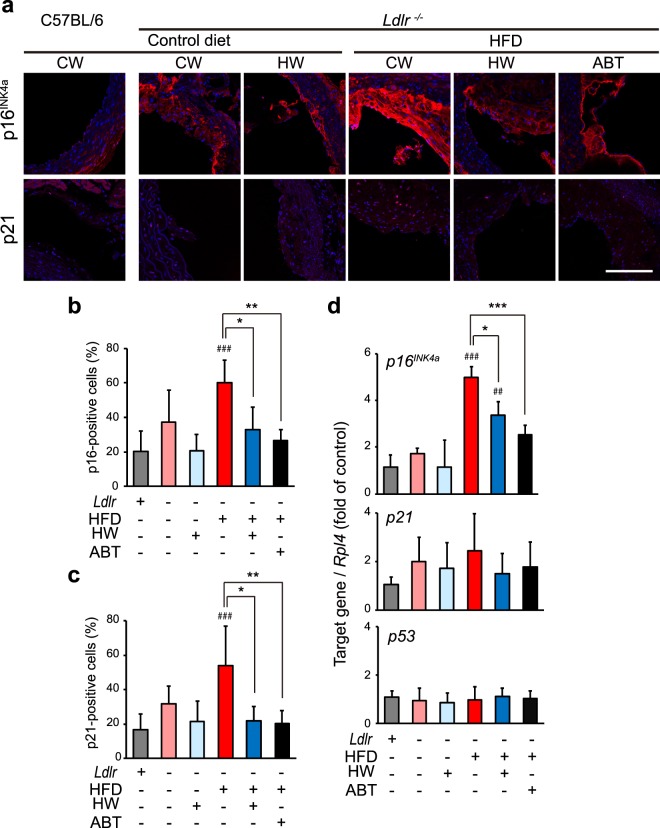
Drinking HW decreased HFD-induced p16^INK4a^ and p21 expression in the ascending aorta in *Ldlr*^−/−^ mice. (**a**) Immunohistochemical staining for p16^INK4a^ and p21 (red) and Hoechst nuclear staining (blue) in the aortic intima. p16^INK4a^- and p21-positive cells were increased in HFD mice given control water (CW). These increases were suppressed in both HFD mice given HW and HFD mice treated with ABT-263 (ABT). Scale bar: 100 μm. (**b**) Quantitative analysis of p16-positive cells out of total cells. (**c**) Quantitative analysis of p21-positive cells out of total cells. (**d**) Transcript levels of cell senescence markers (p16^INK4a^, p21, and p53). Values are the mean ± SD (n = 6). *P < 0.05, **P < 0.01, and ***P < 0.001. ^##^*P* < 0.01 and ^###^*P* < 0.001 vs. C57BL/6 *Ldlr*^+/+^ control mice.

The number of p16^INK4a^-positive cells in the aortic intima was markedly increased in the HFD/control water mice, whereas both drinking HW and oral administration (o.p.) of ABT-263 significantly suppressed this increase (Fig. 2a,b). Similarly, the number of p21-positive cells was markedly increased in the aortic intima in the HFD/control water group, and both HW and ABT-263 significantly suppressed this increase (Fig. 2a,c). To further examine whether cellular senescence was altered by HW, the mRNA levels of cellular senescence markers (p16^INK4a^, p21, p53) in the aortic arch were analyzed by quantitative reverse-transcription PCR (qPCR). Transcript levels of *p16*^*INK4a*^ were significantly increased in all the HFD-fed groups compared with the groups that received a standard diet (Fig. 2d). Both drinking HW and ABT-263 o.p. significantly suppressed the increase in *p16*^*INK4a*^ transcript levels. These results suggest that HW attenuates HFD-induced cellular senescence in the aorta, similarly to ABT-263.

### Drinking HW had little effect on lipid metabolism in HFD-fed *Ldlr*^−/−^ mice

**Figure 3:**
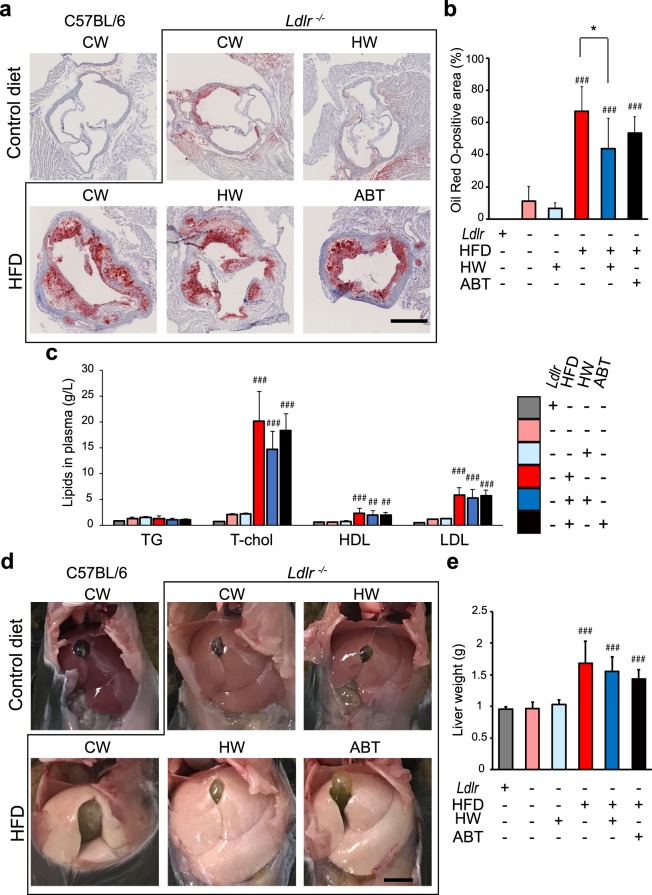
Drinking HW had little effect on lipid metabolism in HFD-fed *Ldlr*^−/−^ mice. (**a**) Oil Red O-positive areas in the vicinity of the aortic valve. The increase in the Oil Red O-positive area in HFD-fed mice given control water (CW) was not suppressed by either HW or ABT-263 (ABT). Scale bar: 500 μm. (**b**) Quantitative analysis of the Oil Red O-positive area in the ascending aorta. (**c**) TG, T-chol, HDL, and LDL levels in the plasma. (**d**) Representative images of fatty liver. Scale bar: 5 mm. (**e**) Quantitative analysis of the liver weight. Values are the mean ± SD (n = 6). *P < 0.05. ^##^*P* < 0.01 and ^###^*P* < 0.001 vs. C57BL/6 *Ldlr*^+/+^ control mice.

Atherosclerosis is induced by the accumulation of oxidized LDL in ECs. Oil Red O is a lysochrome (fat-soluble dye) diazo dye used for staining neutral triglycerides (TGs) and lipids, and conventionally used for measurement of the gross area of the atherosclerotic lesion^18^. The Oil Red O-positive area in the ascending aorta was markedly increased in all the HFD-fed groups, and drinking HW slightly suppressed this increase (Fig. 3a,b), indicating the possibility that lipid metabolism is affected by HW.

TG, total cholesterol (T-chol), high-density lipoprotein (HDL), and LDL levels in the blood plasma were markedly increased in all the HFD-fed groups (Fig. 3c). However, drinking HW did not prevent this increase in lipid levels. Similarly, hepatic steatosis was present in all the HFD-fed groups (Fig. 3d), and drinking HW neither improved the hepatic steatosis nor prevented the increase in liver weight (Fig. 3d,e). These results suggest that drinking HW has little or no effect on lipid metabolism in HFD-fed *Ldlr*^−/−^ mice.

### Drinking HW decreased macrophage infiltration into the aortic intima in HFD-fed *Ldlr*^−/−^ mice

**Figure 4:**
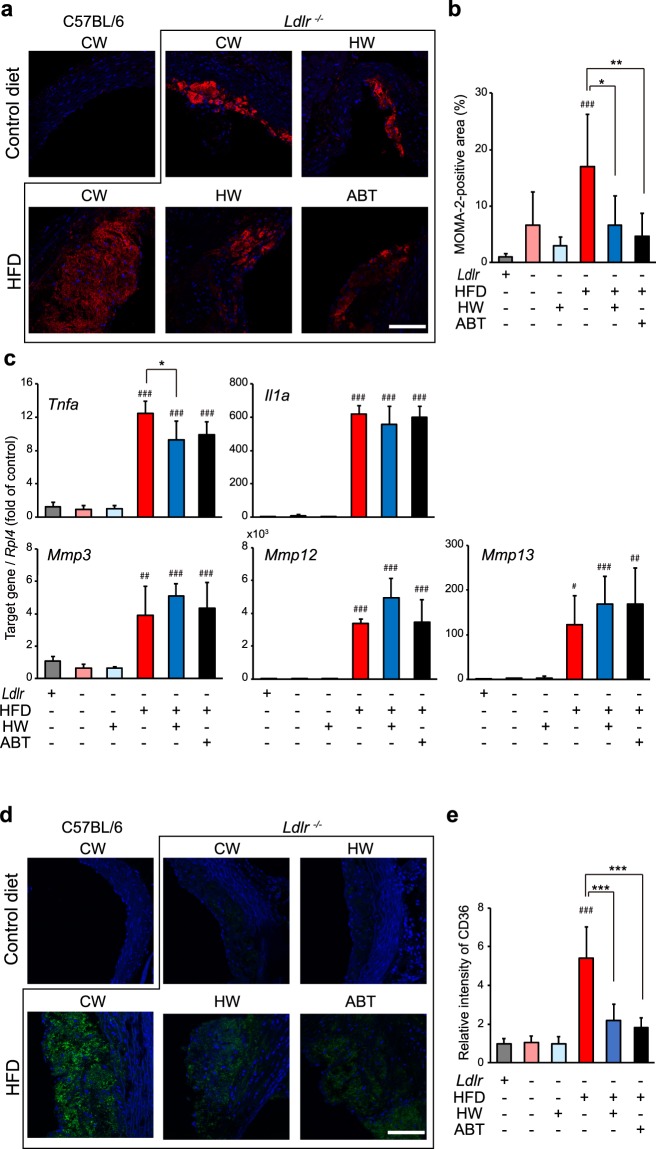
Drinking HW decreased macrophage infiltration into the aortic intima in HFD-fed *Ldlr*^−/−^ mice. (**a**) Immunohistochemical staining for MOMA-2 (red) and Hoechst nuclear staining (blue) in the aortic intima. MOMA-2-positive cells were increased in HFD mice given control water (CW). This increase was suppressed by both HW and ABT-263 (ABT). Scale bar: 100 μm. (**b**) Quantitative analysis of macrophage infiltration in the aortic intima. (**c**) Transcript levels of inflammatory cytokines (*Il1α* and *Tnfα*) and matrix metalloproteinases (*Mmp3*, *Mmp13*, and *Mmp12*). (**d**) Immunohistochemical staining for CD36 (green) and Hoechst nuclear staining (blue) in the aortic intima. CD36-positive cells were increased in HFD mice given control water (CW). This increase was suppressed by both HW and ABT-263 (ABT). Scale bar: 100 μm. (**e**) Quantitative analysis of CD36-positive cells in the aortic intima. Values are the mean ± SD (n = 6). *P < 0.05, **P < 0.01 and ***P < 0.001. ^#^*P* < 0.05, ^##^*P* < 0.01 and ^###^*P* < 0.001 vs. C57BL/6 *Ldlr*^+/+^ control mice.

Foamy macrophages expressing senescence markers accumulate in the subendothelial space and drive atherosclerotic processes by increasing the expression of inflammatory cytokines and matrix metalloproteinases^19,20^. We therefore examined whether the numbers of infiltrating macrophages in the aortic intima were altered by drinking HW. The MOMA-2-positive area in the ascending aorta was markedly larger in the HFD/control water group (Fig. 4a,b). Both HW and ABT-263 significantly reduced the MOMA-2-positive area. To examine whether the expression of inflammatory cytokines and matrix metalloproteinases was altered by HW, their mRNA levels (*Il1α*, *Tnfα*, *Mmp3*, *Mmp13*, *Mmp12*) were measured by qPCR. Their transcript levels were markedly upregulated by HFD (Fig. 4c). Notably, both HW and ABT-263 significantly inhibited the upregulation of *Tnfα*. Recent studies indicate that CD36, a scavenger receptor of oxidized LDL in macrophage^21^, plays an important role in establishing the senescent phenotype^22^. We therefore examined the expression of CD36 in atherosclerotic regions and found that markedly increased level of CD36 in the HFD/control water group was significantly reduced by both HW and ABT-263 (Fig. 4d,e).

### Suppression of cellular senescence by HW correlated with attenuation of macrophage infiltration

**Figure 5:**
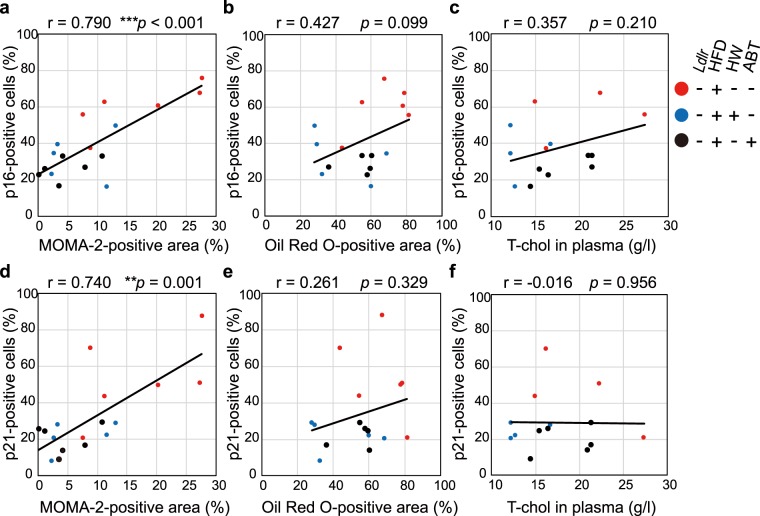
HFD-induced macrophage infiltration correlated with p16^INK4a^/p21 expression. Scatter plots of the number of p16^INK4a^-positive (**a**–**c**) and p21-positive (**d**–**f**) cells versus the MOMA-2-positive (**a**,**d**) and the Oil Red O-positive (**b**,**e**) area in the ascending aorta or versus the T-chol level in the plasma (**c**,**f**). Dots correspond to data from individual mice, as indicated. The Pearson’s correlation coefficient (r) and p-value (*p*) are shown.

Immunohistochemical staining revealed that the increased MOMA-2-positive area in the HFD-fed groups shown in Fig. 4a,b significantly correlated with the numbers of p16^INK4a^- and p21-positive cells shown in Fig. 2a–c (Fig. 5a,d), supporting a strong association between macrophage activation and the development of senescence in the aorta. Both HW and ABT-263 suppressed the infiltration of macrophages and the accumulation of SCs in the aorta. On the other hand, the increased Oil Red O-positive area seen in HFD-fed mice shown in Fig. 3a,b did not correlate with the number of p16^INK4a^-positive or p21-positive cells (Fig. 5b,e), indicating that the effect of lipid accumulation on the induction of SCs was not stronger in the HFD-fed groups. Increased T-chol in the blood plasma also did not correlate with the number of p16^INK4a^- or p21-positive cells (Fig. 5c,f).

## Discussion

The present study demonstrates that drinking HW improves vascular aging in the aorta. Many studies have shown that the cellular senescence markers p16^INK4a^ and p21 are elevated in animal and human atherosclerotic plaques^4,23–25^. We showed that the HFD-induced increase in p16^INK4a^- and p21-positive ECs in the aortic intima (Fig. 1) was significantly suppressed by HW (Fig. 2). Drinking HW also significantly reduced the increase in *p16*^*INK4a*^ transcript levels. Transcript levels of *p21* were not significantly altered but tended to be suppressed by HW. However, *p53* mRNA expression was not affected by any of the treatment conditions. Thus, p21 expression in this model might be regulated by signals other than p53^26^. Furthermore, neither HW nor ABT-263 decreased SA-β-Gal activity in the thoracic aorta in HFD-fed mice (Supplementary Fig. 1). Increase in SA-β-Gal activity is known to be an increase in lysosomal β-Gal activity and is not completely senescence-specific marker^27,28^. The discrepancy may be explained by the differential expression of CDK inhibitors (p16^INK4a^ and p21) and SA-β-Gal. Childs *et al*. reported that foamy macrophages were highly positive for SA-β-Gal in HFD-fed *Ldlr*^−/−^ mice^11^. However, previous studies showed that both p16^INK4a^- and p21-positive cells in atherosclerotic plaques are largely ECs and vascular smooth muscle cells^4,23,24^. Another possible explanation is cellular senescence at vascular branches and bends in *Ldlr*^−/−^ mice^29^. We may have failed to observe the effects of drinking HW by focusing instead on the thoracic aorta, since the accumulation of SCs is lower there.

ROS can induce senescent growth arrest by triggering DNA damage response (DDR) pathways by transcriptional activation of p16^INK4a^ and p21^30^. Activation of these CDK inhibitors generates ROS, which act as signaling molecules to promote the continuous induction of cellular senescence^30^. Remarkably, drinking HW ameliorates oxidative stress in animal disease models, including the *Apoe*^−/−^ mouse model of atherosclerosis^16^, and in human clinical trials, including metabolic syndrome^17,31^ and rheumatoid arthritis^32^. One possible mechanistic explanation for the suppressive effect of HW on cellular senescence is that the lowering of ROS by HW downregulates DDR pathways. We demonstrated previously that H_2_ induces a nuclear factor erythroid 2-related factor 2 (Nrf2)-dependent antioxidant response in cultured cells^33^ and in a mouse model of sepsis^16^. A direct interaction between Nrf2 and p21 upregulates the antioxidant response^34^, which may downregulate further activation of DDR pathways. Interestingly, H_2_-rich medium alleviated dioxin-induced SA-β-Gal-positive cellular senescence in endothelial cells accompanying Nrf2 activation^35^. However, the atheroscrerotic lesions were markedly attenuated in the *Nrf2* and *Apoe* double KO mice after 12 weeks on HFD and significantly amplified by the presence of Nrf2 in bone marrow-derived cells^36^. We then stained the aorta with anti-Nrf2 antibody and found that Nrf2 expression and its nuclear translocation tended to be enhanced by drinking HW in HFD-fed *Ldlr*^−/−^ mice (Supplementary Fig. 2). Nrf2 usually works as an anti-inflammatory factor in various cell types including endotherial cells^37^. Further precise analysis *in vitro* and *in vivo* is needed to investigate whether HW-induced Nrf2 reduces expression of CDK inhibitors by lowering of ROS and inhibits Rb-mediated growth inhibition.

Hyperlipidemic conditions can accelerate atherosclerosis and vascular aging^3,38^. On the other hand, drinking HW ameliorated hyperlipidemia in HFD-fed hamsters^39^ and in patients with potential metabolic syndrome^31^. Administration of hydrogen-saturated saline decreased athero-susceptibility in apoB-containing lipoprotein and aortic atherosclerosis in *Apoe*^−/−^ mice^40^. However, in the present study, HW and ABT-263 had little or no effect on lipid metabolism, with only a limited reduction in the area of the atheroma observed in HFD-fed *Ldlr*^−/−^ mice (Fig. 3). On the other hand, HW significantly reduced an increase of CD36 expression in atherosclerotic region (Fig. 4). CD36 is one of the main scavenger receptors involved in the uptake of oxLDL by macrophages and has generally been viewed as essential for foam cell formation^1^. CD36 expression is known to be induced by oxLDL^41^, suggesting that HW-induced decrease of CD36 expression may depend on the slight suppression of lipid accumulation in the aorta by drinking HW. Another possibility is that H_2_ directly reduces CD36 expression in macrophages. We previously demonstrated that H_2_ inhibits fatty acid uptake and lipid accumulation through the downregulation of CD36 in human hepatoma HepG2 cells^42^.

A range of extrinsic and intrinsic stimuli including inflammatory responses, particularly those causing intracellular oxidative stress, can induce stress-induced premature senescence. In the previous study, atheroscrerotic lesions in *Apo*e KO mice were observed after 12 weeks on HFD diet^3^. Furthermore, elimination of foamy macrophages in *Ldlr*^−/−^ mice prevented atherogenesis onset after 9 days on an atherogenic HFD diet^11^, indicating that atherogenesis after 13 weeks on HFD diet in our model is induced and enhanced by early and continuous inflammatory responses against hyperlipidemia and drinking HW may attenuate them. Indeed, drinking HW significantly suppressed macrophage infiltration and *Tnfα* transcription in the aortic arch of HFD-fed *Ldlr*^−/−^ mice (Fig. 4). Tumor necrosis factor (TNF)-α is primarily produced by activated macrophages and can trigger premature senescence^43^. However, to clarify it, spatiotemporal analyses such as analyzing the atherosclerotic lesion using microdissection or *in situ* hybridization in different ages are necessary. In addition, immunohistochemical staining revealed that the increase in MOMA-2-positive macrophages significantly correlated with the numbers of both p16^INK4a^- and p21-ECs in the same aortic region (Fig. 5).

Several studies reported that H_2_ administration attenuated cellular inflammation and production of inflammatory cytokines in experimental animal models of inflammatory diseases such as zymosan-induced inflammation, inflammatory bowel disease, and lipopolysaccharide (LPS)-induced inflammation^44–46^. H_2_ inhibited LPS-induced phosphorylation of apoptosis signal-related kinase 1 (ASK1) via Toll-like receptors (TLRs)^47^. Signaling via TLRs activates macrophages, resulting in increased secretion of inflammatory cytokines^48,49^. Recent study showed the reduced expression of p16^INK4a^ and p21 in the adipose tissue of TLR4-deficient aged mice^50^, indicating that drinking HW may attenuate cellular senescence by downregulation of TLR signaling. We then stained the aorta with anti-TLR4 antibody and found that drinking HW tended to suppress the increase TLR4 in atheroma, however it was not significant (Supplementary Fig. 3).

The body weight in the ABT-263 group gradually decreased during the course of this study (Supplementary Fig. 4). ABT-263, known as navitoclax, induces apoptosis through its inhibitory effect of the Bcl-2, Bcl-X_L_, and Bcl-W, and it acts at 5 nM in and has high specificity to senescent cells in cell experiments^51^. ABT-263 has been evaluated clinically in a number of trials, both as a monotherapy and in combination with chemotherapy in both solid tumors and hematologic malignancies, with dose-dependent thrombocytopenia as the major adverse effect^52^. On the other hand, any adverse effects of H_2_ administration have not been reported in animal and clinical studies^53^. Here, we showed that drinking HW suppresses cellular senescence in the aorta with no obvious adverse effects. Despite numerous studies reporting the antioxidant and anti-inflammatory effects of H_2_, the molecular mechanisms underlying its actions remain unclear. In the future, it will be important to clarify these mechanisms from the viewpoint of preventing senescence.

## Materials and Methods

### Animals

Female B6.129S7-*Ldlr*^*tm1Her*^*/* J mice (*Ldlr*^−/−^ mice) and female C57BL/6 J mice (specific pathogen-free) were purchased from Charles River Japan, Inc. (Tokyo, Japan). Mice were housed at 20–22 °C with a 12 h light/dark cycle and provided sterile food and water. All efforts were made to minimize the number of animals used and their suffering during experimental procedures. All protocols for animal use followed the Principles of Laboratory Animal Care (NIH publication no. 86-23, revised 1985). All study protocols were reviewed and approved by the Animal Care Committee of the Tokyo Metropolitan Institute of Gerontology.

### HW administration

HW was prepared using a previously described method^17^. In brief, H_2_ gas (G1; Japan fine products, Kawasaki, Japan) was dissolved in reverse osmosis water under high pressure (0.4 MPa) to a super-saturated level in a stainless steel tank (Unicontrols, Chiba, Japan). Saturated HW was poured into aluminum foil bag equipped with an outlet line (Uchida Yoko, Tokyo, Japan) at atmosphere pressure. H_2_ concentration in water was monitored with Clark-type hydrogen microsensor (Unisense, Aarhus N, Denmark). H_2_ concentration in a tap HW ingested by mice at the end of outlet line was 400–700 μM. After drinking, H_2_ concentration in blood and organs was immediately increased and reached approximately 10 μM^13,54^, which was 100–1000 times higher than that of H_2_ (0.1–0.01 μM) produced by enterobacteria from their nutrients including lactulose^15^. Then, H_2_ was quickly exhaled as a gas^15^. During the preparation of HW, the H_2_ concentration in the air was carefully monitored using a H_2_ sensor with an alarm for safety, because H_2_ gas is explosive when the concentration in air is greater than 4%. Water obtained by degassing H_2_ from HW with gentle stirring overnight was used as a control. H_2_ concentration in a control water was no detectable (<1 nM), similarly to a tap water under the Earth’s standard atmosphere containing 0.5 ppm of H_2_. Mice were given water freely, and the vessel was freshly refilled with HW or control water every week. Every time before refill, we monitored H_2_ concentration in a tap HW used for a week and confirmed that it was approximately 400 μM or higher.

### Experimental design

To induce atherosclerosis, *Ldlr*^−/−^ mice were fed a HFD consisting of 36% calories from fat (H2HFD1; Oriental Yeast, Tokyo, Japan). To determine whether HW eliminates SCs, 10-week-old *Ldlr*^−/−^ mice (n = 30) were randomly divided into five groups: 1) regular chow diet (control diet) with control water (n = 6); 2) control diet with HW (n = 6); 3) HFD with control water (n = 6); 4) HFD with HW (n = 6); and 5) HFD with control water and 50 mg/kg ABT-263 o.p. (Selleck Chemicals, Houston, TX, USA) in vehicle (corn oil with 10% dimethyl sulfoxide) once every 2 days during weeks 5–7 and 10–12 (n = 6). The dose of ABT-263 was referred to previous studies^55^. Female C57BL/6 J mice fed a control diet and provided control water in the same facility for the same term were used as an additional control.

After overnight fasting, mice were sacrificed at 13 weeks from the start of the experiment via exsanguination under deep anesthesia with combined anesthetic agents according to a previously described protocol^16^. In brief, three different anesthetic agents [0.75 mg/kg medetomidine hydrochloride (Domitol; Meiji Seika Pharma, Tokyo, Japan), 4 mg/kg midazolam (Dormicum; Astellas Pharma, Tokyo, Japan), and 5 mg/kg butorphanol (Vetorphale; Meiji Seika Pharma)] were mixed and administered to the mice by intraperitoneal injection. Plasma was isolated from heparinized blood by centrifugation at 2000 × *g* for 10 min at room temperature.

### Analyses of plasma lipids

Plasma TG and T-chol levels were determined with enzymatic colorimetric methods according to the manufacturer’s protocol (Wako, Osaka, Japan). HDL and LDL levels were determined with enzymatic colorimetric methods according to the manufacturer’s protocol (Nittobo, Tokyo, Japan).

### Oil Red O staining

Atheromatous plaques in the aorta were fixed in 4% paraformaldehyde, cryoprotected with sucrose, frozen, and cut into 8 μm sections. Sections were incubated with Oil Red O (Muto, Tokyo, Japan) for 15 min at 37 °C. After washing, sections were counterstained with hematoxylin (Wako) for 10 min at room temperature. The sections were scanned and observed on a whole slide scanner (Nanozoomer; Hamamatsu Photonics, Hamamatsu, Japan).

### Immunohistochemistry

Aortas were fixed with 4% paraformaldehyde (Wako), cryoprotected with sucrose, frozen, and cut into 8 μm sections. Each section was blocked with goat serum and incubated overnight at 4 °C with primary antibodies against mouse antigens, as follows: monoclonal mouse anti-p16^INK4a^ (1:200; Abcam, Cambridge, UK), monoclonal rabbit anti-p21(1:500; Abcam), monoclonal rat anti-CD31 (1:200; Abcam), MOMA-2 (1:25; Bio-Rad, Hercules, CA, USA), polyclonal goat anti-SMA (1:300; Abcam), monoclonal rabbit anti-CD36 (1:200; Abcam), monoclonal mouse anti-TLR4 (1:200; Abcam) and polyclonal rabbit anti-Nrf2 (1:200; C-20, Santa Cruz, Dallas, TX, USA). After washing, sections were further incubated with the appropriate secondary antibodies [Alexa Fluor 546-labeled goat anti-mouse IgG (1:500; ThermoFisher Scientific, Waltham, MA, USA), Alexa Fluor 546-labeled goat anti-rabbit IgG (1:500; ThermoFisher), Alexa Fluor 488-labeled goat anti-rat IgG (1:400; ThermoFisher), Alexa Fluor 594-labeled goat anti-rat IgG (1:400; ThermoFisher), Alexa Fluor 488-labeled donkey anti-goat IgG (1:500; ThermoFisher), Alexa Fluor 546-labeled donkey anti-mouse IgG (1:500; ThermoFisher), or Alexa Fluor 546-labeled donkey anti-rabbit IgG (1:500; ThermoFisher)] for 60 min at room temperature. The aortas were counterstained with Hoechst 33342 (1:2000; Nacalai, Tokyo, Japan) and imaged on a TCS SP8 laser confocal microscope (Leica, Wetzlar, Germany).

### qPCR of senescence-associated genes

The fresh-frozen aortic arches were used for quantitative PCR analysis. Their total cellular RNA was extracted using the NucleoSpin kit (Macherey-Nagel, Düren, Germany) and subjected to reverse transcription using a first-strand synthesis system (SuperScript II; ThermoFisher Scientific) according to the manufacturer’s protocol. qPCR was performed using SYBR Green (Toyobo, Osaka, Japan) according to the manufacturer’s recommendations, and target gene expression was normalized to the expression of *Rpl4* in each sample. The primers used for amplification of *p16*^*INK4a*^, *p19*^*Arf*^, *p21*, *p53*, *Il1α*, *Tnfα*, *Mmp3*, *Mmp13*, *Mmp12*, and *Rpl4* cDNAs are listed in Table 1.

**Table 1:** Primers used for semiquantitative PCR.

Gene	Forward (F)/Reverse (R)	Primer sequences
*Il1α*	F	ACACTATCTCAGCACCACTTGG
	R	CGCTCACGAACAGTTGTGAATC
*Tnfa*	F	TCCCAAATGGCCTCCCTCTC
	R	TGCTCCTCCACTTGGTGGTTT
*MMP3*	F	GACTCAAGGGTGGATGCTGTC
	R	TTGGGTCAAATTCCAACTGCGA
*MMP12*	F	AGAGGTCAAGATGGATGAAGCG
	R	GAGTCACATCACTCCAGACTTGG
*MMP13*	F	CACTGGCAAAAGCCATTTCATGC
	R	TGCTTAGGGTTGGGGTCTTCATC
*p16INK4a*	F	GTGTGCATGACGTGCGGG
	R	ACGTGAACGTTGCCCATCATC
*p21*	F	GTGGCCTTGTCGCTGTCTTG
	R	CCAATCTGCGCTTGGAGTGATAG
*p53*	F	CCTCTGAGCCAGGAGACATTTTC
	R	AACAGATCGTCCATGCAGTGAG
*RPL4 (internal control)*	F	GCCAAGACTATGCGCAGGAAT
	R	GTAGCTGCTGCTTCCAGCTT

### Statistical analysis

Statistical analyses were performed using SPSS software (version 22.0; SPSS, Chicago, IL, USA). All values are presented as the means ± standard deviation (SEM). Significance was obtained by one-way ANOVA with Tukey’s test. The Pearson’s correlation coefficient was used to test correlations. Results were considered significant at *P* < 0.05.

## Electronic supplementary material

Supplementary Information

## References

[1] Weber C, Noels H (2011). Atherosclerosis: current pathogenesis and therapeutic options. Nat Med.

[2] Freigang S (2013). Fatty acid-induced mitochondrial uncoupling elicits inflammasome-independent IL-1alpha and sterile vascular inflammation in atherosclerosis. Nat Immunol.

[3] Wang JC, Bennett M (2012). Aging and atherosclerosis: mechanisms, functional consequences, and potential therapeutics for cellular senescence. Circ Res.

[4] Holdt LM (2011). Expression of Chr9p21 genes CDKN2B (p15(INK4b)), CDKN2A (p16(INK4a), p14(ARF)) and MTAP in human atherosclerotic plaque. Atherosclerosis.

[5] Rossman MJ (2017). Endothelial cell senescence with aging in healthy humans: prevention by habitual exercise and relation to vascular endothelial function. Am J Physiol Heart Circ Physiol.

[6] Coppe JP (2008). Senescence-associated secretory phenotypes reveal cell-nonautonomous functions of oncogenic RAS and the p53 tumor suppressor. PLoS Biol.

[7] Rodier F (2009). Persistent DNA damage signalling triggers senescence-associated inflammatory cytokine secretion. Nat Cell Biol.

[8] Childs BG, Durik M, Baker DJ, Van Deursen JM (2015). Cellular senescence in aging and age-related disease: from mechanisms to therapy. Nat Med.

[9] Munoz-Espin D, Serrano M (2014). Cellular senescence: from physiology to pathology. Nat Rev Mol Cell Biol.

[10] Kuilman T, Michaloglou C, Mooi WJ, Peeper DS (2010). The essence of senescence. Genes Dev.

[11] Childs BG (2016). Senescent intimal foam cells are deleterious at all stages of atherosclerosis. Science.

[12] Ohsawa I (2007). Hydrogen acts as a therapeutic antioxidant by selectively reducing cytotoxic oxygen radicals. Nat Med.

[13] Iketani M, Ohsawa I (2017). Molecular Hydrogen as a Neuroprotective Agent. Curr Neuropharmacol.

[14] Ichihara M (2015). Beneficial biological effects and the underlying mechanisms of molecular hydrogen - comprehensive review of 321 original articles. Med Gas Res.

[15] Ito M (2012). Drinking hydrogen water and intermittent hydrogen gas exposure, but not lactulose or continuous hydrogen gas exposure, prevent 6-hydorxydopamine-induced Parkinson’s disease in rats. Med Gas Res.

[16] Iketani M (2017). Preadministration of Hydrogen-Rich Water Protects Against Lipopolysaccharide-Induced Sepsis and Attenuates Liver Injury. Shock.

[17] Ohsawa I, Nishimaki K, Yamagata K, Ishikawa M, Ohta S (2008). Consumption of hydrogen water prevents atherosclerosis in apolipoprotein E knockout mice. Biochem Biophys Res Commun.

[18] Maganto-Garcia, E., Tarrio, M. & Lichtman, A. H. Mouse models of atherosclerosis. *Curr Protoc Immuno*l Chapter 15, Unit15 24 11–23, 10.1002/0471142735.im1524s96 (2012). 10.1002/0471142735.im1524s96 22314832

[19] Johnson JL (2017). Metalloproteinases in atherosclerosis. Eur J Pharmacol.

[20] Ramji DP, Davies TS (2015). Cytokines in atherosclerosis: Key players in all stages of disease and promising therapeutic targets. Cytokine Growth Factor Rev.

[21] Endemann G (1993). CD36 is a receptor for oxidized low density lipoprotein. J Biol Chem.

[22] Chong Mengyang, Yin Tao, Chen Rui, Xiang Handan, Yuan Lifeng, Ding Yi, Pan Christopher C, Tang Zhen, Alexander Peter B, Li Qi‐Jing, Wang Xiao‐Fan (2018). CD36 initiates the secretory phenotype during the establishment of cellular senescence. EMBO reports.

[23] Tanner FC (1998). Expression of cyclin-dependent kinase inhibitors in vascular disease. Circ Res.

[24] Matthews C (2006). Vascular smooth muscle cells undergo telomere-based senescence in human atherosclerosis: effects of telomerase and oxidative stress. Circ Res.

[25] Motterle A (2012). Functional analyses of coronary artery disease associated variation on chromosome 9p21 in vascular smooth muscle cells. Hum Mol Genet.

[26] Abbas T, Dutta A (2009). p21 in cancer: intricate networks and multiple activities. Nat Rev Cancer.

[27] Lee BY (2006). Senescence-associated beta-galactosidase is lysosomal beta-galactosidase. Aging Cell.

[28] Debacq-Chainiaux F, Erusalimsky JD, Campisi J, Toussaint O (2009). Protocols to detect senescence-associated beta-galactosidase (SA-betagal) activity, a biomarker of senescent cells in culture and *in vivo*. Nat Protoc.

[29] Warboys CM (2014). Disturbed flow promotes endothelial senescence via a p53-dependent pathway. Arterioscler Thromb Vasc Biol.

[30] Davalli P, Mitic T, Caporali A, Lauriola A, D’Arca D (2016). ROS, Cell Senescence, and Novel Molecular Mechanisms in Aging and Age-Related Diseases. Oxid Med Cell Longev.

[31] Song G (2013). Hydrogen-rich water decreases serum LDL-cholesterol levels and improves HDL function in patients with potential metabolic syndrome. J Lipid Res.

[32] Ishibashi T (2012). Consumption of water containing a high concentration of molecular hydrogen reduces oxidative stress and disease activity in patients with rheumatoid arthritis: an open-label pilot study. Med Gas Res.

[33] Murakami Y, Ito M, Ohsawa I (2017). Molecular hydrogen protects against oxidative stress-induced SH-SY5Y neuroblastoma cell death through the process of mitohormesis. PLoS One.

[34] Chen W (2009). Direct interaction between Nrf2 andp21(Cip1/WAF1) upregulates the Nrf2-mediated antioxidant response. Mol Cell.

[35] Hara F (2016). Molecular Hydrogen Alleviates Cellular Senescence in Endothelial Cells. Circ J.

[36] Harada N (2012). Nrf2 in bone marrow-derived cells positively contributes to the advanced stage of atherosclerotic plaque formation. Free Radic Biol Med.

[37] Kloska, D. *et al*. Nrf2 in aging - Focus on the cardiovascular system. *Vascul Pharmacol*, 10.1016/j.vph.2018.08.009 (2018). 10.1016/j.vph.2018.08.009 30170173

[38] Katsuumi G, Shimizu I, Yoshida Y, Minamino T (2018). Vascular Senescence in Cardiovascular and Metabolic Diseases. Front Cardiovasc Med.

[39] Zong C (2012). Administration of hydrogen-saturated saline decreases plasma low-density lipoprotein cholesterol levels and improves high-density lipoprotein function in high-fat diet-fed hamsters. Metabolism.

[40] Song G (2012). Hydrogen decreases athero-susceptibility in apolipoprotein B-containing lipoproteins and aorta of apolipoprotein E knockout mice. Atherosclerosis.

[41] Feng J (2000). Induction of CD36 expression by oxidized LDL and IL-4 by a common signaling pathway dependent on protein kinase C and PPAR-gamma. J Lipid Res.

[42] Iio A (2013). Molecular hydrogen attenuates fatty acid uptake and lipid accumulation through downregulating CD36 expression in HepG2 cells. Med Gas Res.

[43] Zhang Y (2009). Premature senescence of highly proliferative endothelial progenitor cells is induced by tumor necrosis factor-alpha via the p38 mitogen-activated protein kinase pathway. FASEB J.

[44] Xie K (2010). Hydrogen gas improves survival rate and organ damage in zymosan-induced generalized inflammation model. Shock.

[45] Xie K (2012). Molecular hydrogen ameliorates lipopolysaccharide-induced acute lung injury in mice through reducing inflammation and apoptosis. Shock.

[46] Qiu X (2011). Hydrogen inhalation ameliorates lipopolysaccharide-induced acute lung injury in mice. Int Immunopharmacol.

[47] Itoh T (2011). Molecular hydrogen inhibits lipopolysaccharide/interferon gamma-induced nitric oxide production through modulation of signal transduction in macrophages. Biochem Biophys Res Commun.

[48] Newby AC (2016). Metalloproteinase production from macrophages - a perfect storm leading to atherosclerotic plaque rupture and myocardial infarction. Exp Physiol.

[49] Lundberg AM, Hansson GK (2010). Innate immune signals in atherosclerosis. Clin Immunol.

[50] Ghosh AK, O’Brien M, Mau T, Yung R (2017). Toll-like receptor 4 (TLR4) deficient mice are protected from adipose tissue inflammation in aging. Aging (Albany NY).

[51] Zhu Y (2016). Identification of a novel senolytic agent, navitoclax, targeting the Bcl-2 family of anti-apoptotic factors. Aging Cell.

[52] Gandhi L (2011). Phase I study of Navitoclax (ABT-263), a novel Bcl-2 family inhibitor, in patients with small-cell lung cancer and other solid tumors. J Clin Oncol.

[53] Ohta S (2014). Molecular hydrogen as a preventive and therapeutic medical gas: initiation, development and potential of hydrogen medicine. Pharmacol Ther.

[54] Nakashima-Kamimura N, Mori T, Ohsawa I, Asoh S, Ohta S (2009). Molecular hydrogen alleviates nephrotoxicity induced by an anti-cancer drug cisplatin without compromising anti-tumor activity in mice. Cancer Chemother Pharmacol.

[55] Chang J (2016). Clearance of senescent cells by ABT263 rejuvenates aged hematopoietic stem cells in mice. Nat Med.

