# Supplementary material for: Administration of hydrogen-rich water prevents vascular aging of the aorta in LDL receptor-deficient mice
Source: Sci Rep. 2018 Nov 14;8:16822. doi: 10.1038/s41598-018-35239-0 (PMC6235982; doi:10.1038/s41598-018-35239-0)
Supplement: Supplementary file 1 — Supplementary Information [file 41598_2018_35239_MOESM1_ESM.pdf]

Supplementary Information

Administration of hydrogen-rich water prevents vascular aging of the aorta in LDL receptor-deficient mice

Masumi Iketani[1], Kanako Sekimoto[1,2], Tsutomu Igarashi[3], Mayumi Takahashi[1], Masaki Komatsu[1,2], Iwao Sakane[1,4], Hiroshi Takahashi[3], Hideo Kawaguchi[2], Ritsuko Ohtani-Kaneko[2], Ikuroh Ohsawa[1*]

[1]Biological Process of Aging, Tokyo Metropolitan Institute of Gerontology, Tokyo, Japan

[2]Department of Life Sciences, Toyo University, Gunma, Japan

[3]Department of Ophthalmology, Nippon Medical School, Tokyo, Japan

[4]Central Research Institute, ITO EN Ltd., Makinohara, Japan

*Corresponding author: iohsawa@tmig.or.jp

Supplementary Methods

Supplementary Figures 1-4

Supplementary Methods

SA-β-Gal assay. SA-β-Gal staining of aortas was performed according to the manufacturer's instructions (Cell Signaling, Danvers, MA, USA). Mouse thoracic aortas were excised and stored in phosphate-buffered saline on ice until fixation. Aortas were fixed with 1 × Fixative Solution for 15 min at room temperature, washed twice with phosphate-buffered saline, and incubated overnight in staining solution at 37 °C.

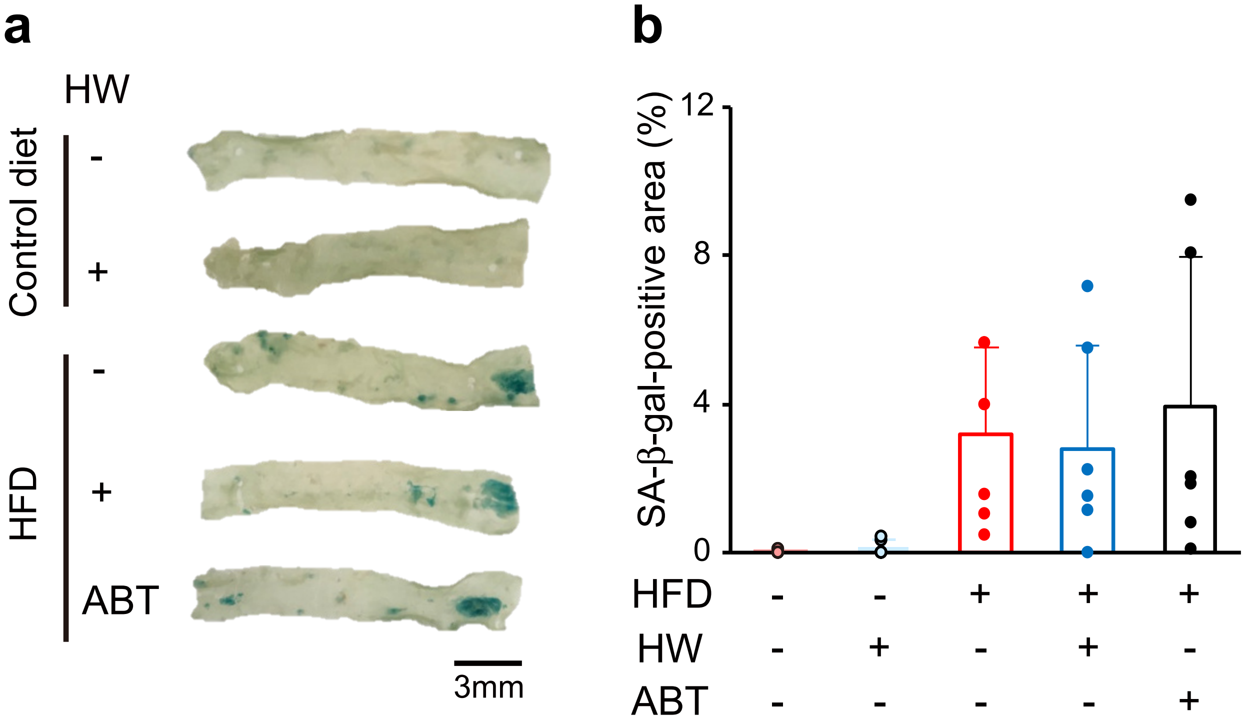

Supplementary Figure 1. Drinking HW did not decrease aortic SA-β-gal staining in *Ldlr*$^{-/-}$ mice. (**a**) The SA-β-gal-positive area in the thoracic aorta was increased in the HFD-fed groups. (**b**) Quantitative analysis of the SA-β-gal-positive area in the aorta. Neither HW nor ABT-263 (ABT) suppressed the increase in the SA-β-gal-positive area. Values are the mean ± SD (n = 6).

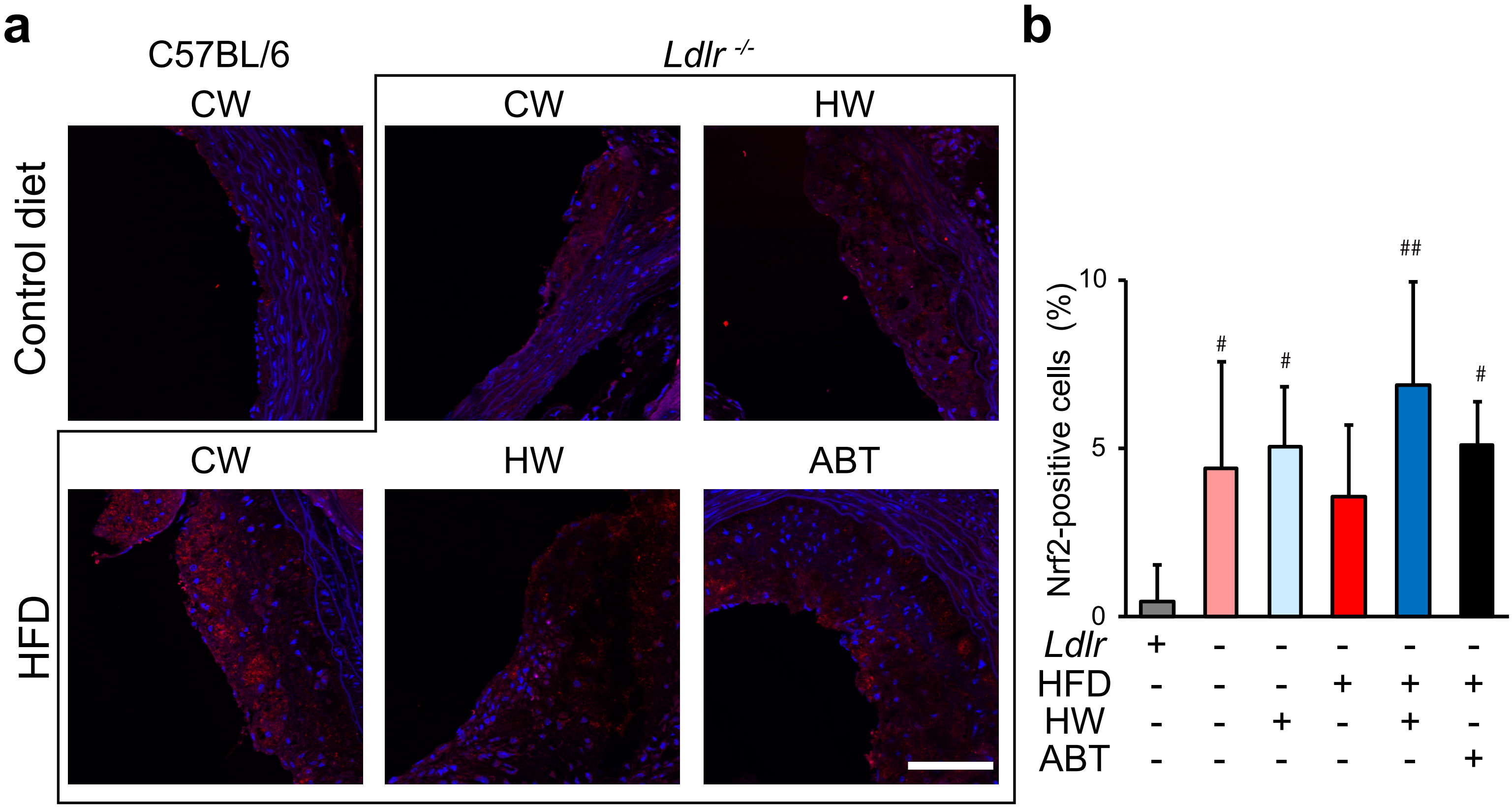

Supplementary Figure 2. Drinking HW increased Nrf2 positive cells in the aorta of HFD-fed *Ldlr*$^{-/-}$ mice. (**a**) Immunohistochemical staining for Nrf2 (red) and Hoechst nuclear staining (blue) in the aortic intima. Nrf2-positive cells were increased in HFD mice given control water (CW). This increase was further enhanced by both HW and ABT-263 (ABT). Scale bar: 100 µm. (**b**) Quantitative analysis of Nrf2-positive cells in the aortic intima. $^{\#}P < 0.05$ and $^{\#\#}P < 0.01$ vs. C57BL/6 *Ldlr*$^{+/+}$ control mice.

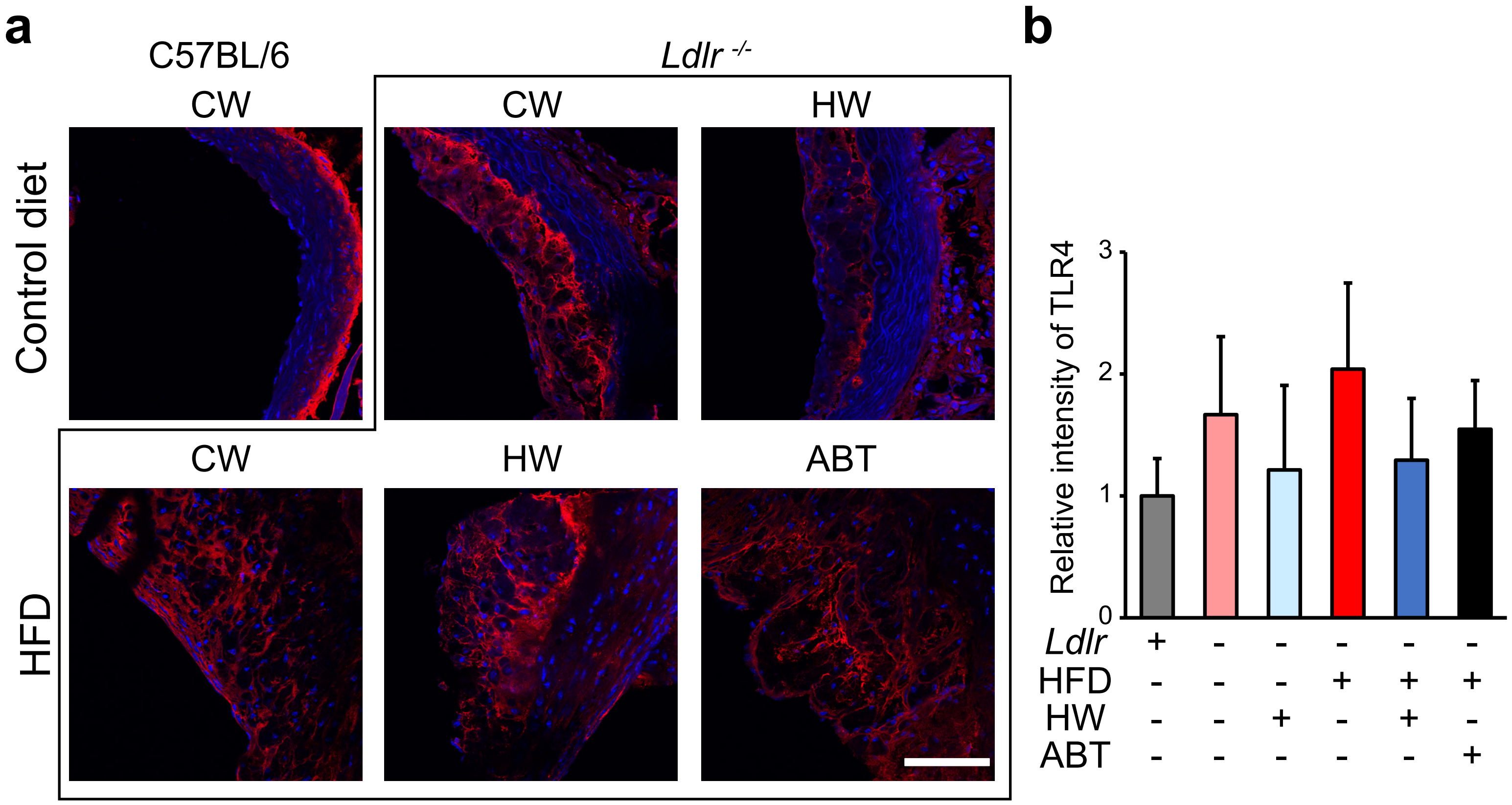

Supplementary Figure 3. Effect of drinking HW on the expression of TLR4 in the aorta of HFD-fed *Ldlr*$^{-/-}$ mice. (**a**) Immunohistochemical staining for TLR4 (red) and Hoechst nuclear staining (blue) in the aortic intima. Scale bar: 100 μm. (**b**) Quantitative analysis of TLR4-intensity in the aortic intima.

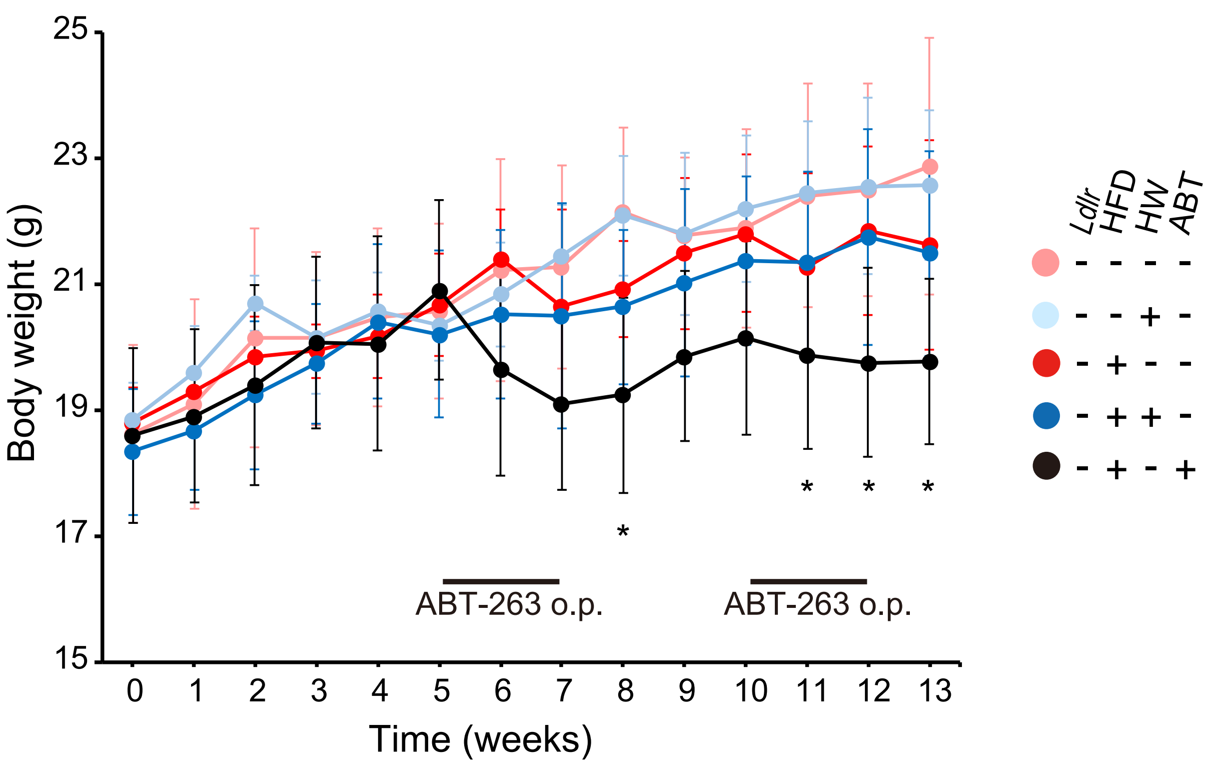

Supplementary Figure 4. Weight gain in *Ldlr*$^{-/-}$ mice was prevented by ABT-263. Values are the mean ± SD (n = 6). *$P < 0.05$.
